# Modeling the impact of early interventions on the transmission dynamics of coronavirus infection

**DOI:** 10.12688/f1000research.54268.1

**Published:** 2021-06-30

**Authors:** Christopher Saaha Bornaa, Baba Seidu, Yakubu Ibrahim Seini

**Affiliations:** 1 Department of Mathematics and ICT Education,, School of Science, Mathematics and Technology Education, CK Tedam University of Technology and Applied Sciences, Navrongo, UER, +233, Ghana; 2 Department of Mathematics, Faculty of Mathematical sciences, CK Tedam University of Technology and Applied Sciences,, Navrongo, UER, +233, Ghana; 3 Department of Engineering, School of Engineering, University for Development Studies,, Tamale,, UER, +233, Ghana

**Keywords:** Coronavirus, stability, simulation, reproduction number, interventions, transmission

## Abstract

A deterministic model is proposed to describe the transmission dynamics of coronavirus infection with early interventions. Epidemiological studies have employed modeling to unravel knowledge that transformed the lives of families, communities, nations and the entire globe. The study established the stability of both disease free and endemic equilibria. Stability occurs when the reproduction number, R0, is less than unity for both disease free and endemic equilibrium points. The global stability of the disease-free equilibrium point of the model is established whenever the basic reproduction number R0 is less than or equal to unity. The reproduction number is also shown to be directly related to the transmission probability (β), rate at which latently infected individuals join the infected class (δ) and rate of recruitment (Λ). It is inversely related to natural death rate (μ), rate of early treatment (τ _1_), rate of hospitalization of infected individuals (θ) and Covid-induced death rate (σ). The analytical results established are confirmed by numerical simulation of the model.

## Introduction

Accurate detection of the underlying agent that causes a disease is very important. Globally, many infections remain undetected and therefore untreated, causing complications and major human and economic consequences. Managing diseases in our modern world requires strategic interventions at particular points during the infection cycle. These are before the infection, early stages of the infection (asymptomatic) and later stages of the infection (symptomatic). Before the infection, individuals may be immunized as a control measure. Immunization is a cost-effective and highly successful health intervention used to manage emerging infectious diseases such as coronavirus. This is a process where a person, through vaccination, becomes protected against an emerging disease. Early treatment of the disease can also be a good strategy in managing infectious diseases. It is therefore important to identify infected persons early enough to put them on treatment. Early treatment of SARS-CoV-2 would speed up the recovery process, reduce the likelihood of developing severe outcomes and reduce demand on health care systems [ Kim et al., 2020; Strain et al., 2005]. Strain et al. [2005] studied the effect of early treatment of primary infection on cellular reservoir clearance of HIV-1 and noted a positive effect. The question arises of whether this will be the case for coronavirus infections. Individuals should also be encouraged to go for voluntary testing as this will increase case detection, thereby reducing the number of secondary infections [ Kim et al., 2020]. In every infection, the exposure of an individual to the source of infection is followed by an incubation period which varies from one infectious disease to the other. For example, the incubation period for coronavirus disease 2019 (Covid-19) is 14 days. During the incubation period the infected individual may be asymptomatic and unaware of their infection. This is followed by the onset of symptoms and varied health behaviours of the infected individual. Some researchers support the notion that antiviral therapy hastens viral clearance in asymptomatic infection [ Strain et al., 2005; Hu et al., 2020] thereby halting the progression to symptomatic infection and the subsequent impact on the health system and the society at large.

Isolating persons who have been exposed to a coronavirus disease in order to prevent transmission has been a long established public health strategy [ Kim et al., 2020]. This is done either in isolation homes or at hospitals in severe cases. Treatment interventions are offered in such circumstances.

This paper looks at the effect of early interventions on the transmission dynamics of coronavirus infection by employing mathematical modelling. Several mathematical models have been used as an engine to unravel knowledge in many epidemiological studies [ Asamoah et al., 2017, 2020a, 2020b; Bornaa et al., 2017, 2020; Seidu et al., 2020; Agusto et al., 2015; Ivorra Benjamin and Ramos, 2015]. Most of these studies have yielded exceptional results that transformed the lives of families, communities, nations and the entire globe. Mathematical models have also been used to study pest and worm infestation in agriculture and aquaculture. Mathematical modeling is used in economics to observe, understand, and make predictions about human economic behavior.

## Model formulation

Consider *N*( *t*) as the size of the total population at time *t.* The population is then subdivided into five classes: Susceptible, *S*( *t*), the latently infected (Exposed) *E*( *t*), the clinically symptomatic (Infected), *I*( *t*), the hospitalized in a facility (Hospitalized) *H*( *t*) and Recovered *R*( *t*) Classes. Thus, the population is given as

N(t)=S(t)+E(t)+I(t)+H(t)+R(t).

People are recruited into the susceptible population through birth and immigration at rate Λ and leave by having active contact with the viral source and being infected at rate *β.* These individuals progress to the latently infected class *E*( *t*). The susceptible population also decreases by natural death at the rate *μ.* Thus;

$$\frac{dS}{dt}=\Lambda -\left(\beta \;I+\mu \right)\;S.$$

The population of the latently infected (exposed) class decreases whenever an exposed individual begins to show clinical symptoms and is moved into the infected class *I*( *t*) at rate *δ.* This may be due to lack of intervention or intervention failure. It also decreases through natural and disease induced deaths, and recovery from early intervention (treatment) at rates *μ*, *σ* and *τ*_1_ respectively. Thus;

$$\frac{dE}{dt}=\beta \;S\;I-\left(\tau_{1}+\delta +\sigma +\mu \right)\;E.$$

The population of clinically infected individuals decreases due to hospitalization, natural death and disease induced death at rates *θ*, *μ* and *σ* respectively. Thus;

$$\frac{dI}{dt}=\delta \;E-\left(\theta +\sigma +\mu \right)\;I.$$

The population of the individuals that are hospitalized increases whenever the clinically infected individuals are taken to the hospital at rate *θ*, then decreases due to natural death, disease induced death and recovery at rate *τ*_2_. Thus;

$$\frac{dH}{dt}=\theta \;I-\left(\tau_{2}+\sigma +\mu \right)\;H.$$

The population of the recovered individuals increases whenever there is recovery from the exposed class due to early treatment, and also from hospitalized individuals due to recovery after treatment at rates *τ*_1_ and *τ*_2_ respectively, and decreases by natural death. For the period under consideration, the recovered is assumed to have permanent immunity.

$$\frac{dR}{dt}=\tau_{1}\;E+\tau_{2}\;H-\mu \;R.$$

The description of the dynamics of the disease infection is depicted by following set of equations.

$$\left.\begin{array}{l} \frac{dS}{dt}=\Lambda -\left(\beta \;I+\mu \right)\;S\\ \frac{dE}{dt}=\beta \;S\;I-\left(\tau_{1}+\delta +\sigma +\mu \right)\;E\\ \frac{dI}{dt}=\delta \;E-\left(\theta +\sigma +\mu \right)\;I\\ \frac{dH}{dt}=\theta \;I-\left(\tau_{2}+\sigma +\mu \right)\;H\\ \frac{dR}{dt}=\tau_{1}\;E+\tau_{2}\;H-\mu \;R\\ \left(S(0),\;E(0),\;I(0),\;H(0),\;R(0)\right)\in R_{\geq 0}^{5} \end{array}\right\}$$ (1)

For purposes of analysis, let *k*_1_ = *τ*_1_ + *δ* + *σ* + *μ*, *k*_2_ = *θ* + *σ* + *μ*, and *k*_3_ = *τ*_2_ + *σ* + *μ.*

We begin to discuss some basic properties of model ( 1) in the next section. This includes the positivity and boundedness, equilibrium states, local and global stability of the equilibria and sensitivity analysis. Numerical simulation is also employed to confirm the analytical results. Finally, the results are discussed and are conclusions drawn.

## Qualitative properties of the model

### Positivity and boundedness

**Lemma 0.1.***If only all solutions of model ( 1) start in *Ω*, they remain in it for allt* ≥ 0.

*Also, for the model ( 1), the region* $\Omega =\left\{\left.\left(S,\;E,\;I,\;H,\;R\right)\in R_{\geq 0}^{5}\right|N\leq \frac{\Lambda }{\mu }\right\}$ *is a positively invariant set.*

*Proof.* Define $\zeta (x)=\left\{x(t)=0\;\text{ and}\;\left(S,\;E,\;I,\;H,\;R\right)\in \;R_{\geq 0}^{5}\right\}$, $\forall \;x\in \left\{S,\;E,\;I,\;H,\;R\right\}$.

The following therefore is obtained from model ( 1);

$$\left\{\begin{array}{l} {\left.\frac{dS}{dt}\right|}_{\zeta (S)}=\Lambda >0\\ {\left.\frac{dE}{dt}\right|}_{\zeta (E}=\beta \;I\;S>0\\ {\left.\frac{dI}{dt}\right|}_{\zeta (I)}=\delta \;E>0\\ {\left.\frac{dH}{dt}\right|}_{\zeta (H)}=\theta \;I>0\\ {\left.\frac{dR}{dt}\right|}_{\zeta (R)}=\left(\tau_{1}\;E+\tau_{2}\;H\right)>0 \end{array}\right.$$

As stated in Lemma 2 of Yang et al. [1996], a solution of model ( 1) is always such that $\left(S(t),\;E(t),\;I(t),\;H(t),\;R(t)\right)\in R_{\geq 0}^{5}$. The first part of the lemma is therefore proved.

We have

$$\frac{dN}{dt}=\Lambda -N\mu -\left(I+H+E\right)\sigma \leq \Lambda -\mu \;N$$

from *N* = *S* + + *E* + *I* + *H* + *R.* Thus, $N(t)\leq N(0)e^{-\mu \;t}+\frac{\Lambda }{\mu }\left(1-e^{-\mu }\right)$.

Therefore if $0\leq N(0)\leq \frac{\Lambda }{\mu }$ then $\leq \underset{t\to \infty }{\mathrm{limsup}}N(t)\leq \frac{\Lambda }{\mu }$.

Thus, all solutions of the model remain in Ω whenever they start in it. The second part of the lemma is also proved and hence the whole Lemma is proved.

We therefore conclude that model ( 1) is mathematically and epidemiologically well-posed within Ω [ Hethcote, 2000].

### Equilibrium points of the model

There are basically two equilibria for model ( 1); the disease-free, $E_{0}$ and endemic, $E^{*}$ equilibria. The solution of the system

$$\frac{dS}{dt}=\frac{dE}{dt}=\frac{dI}{dt}=\frac{dH}{dt}=\frac{dR}{dt}=0$$

gives the equilibrium points of the model.

Assume that an equilibrium point of the model is typically $\left(S^{*},\;E^{*},\;I^{*},\;H^{*},R^{*}\right)$, then model ( 1) gives;

$$\left.\begin{array}{l} S^{*}=\frac{\Lambda }{\beta \;I+\mu },\\ E^{*}=\frac{\beta \;\Lambda \;I}{\left(\beta \;I+\mu \right)k_{1}},\\ I^{*}=\frac{\delta \;\beta \;\Lambda -\mu \;k_{1}k_{2}}{\beta }\;\text{or}\;0\\ H^{*}=\frac{\theta \;I}{k_{3}},\\ R^{*}=\frac{\left(\tau_{1}\beta \;\Lambda \;k_{3}+\tau_{2}\theta \;\left(\beta \;I+\mu \right)k_{1}\right)I}{\left(\beta \;I+\mu \right)k_{1}\mu }. \end{array}\right\}$$ (2)

**Theorem 0.1.** *The model (* 1 *) has unique endemic and disease free equilibria.*

*Proof.* The solution of 2 for *I* ^*^ = 0 is $E_{0}=\left(S_{0},\;0,\;0,\;0,\;0\right)$, where $S_{0}=\frac{\Lambda }{\mu }$. When $I^{*}=\frac{\delta \;\beta \;\Lambda -\mu \;k_{1}k_{2}}{\beta }$ is substituted into ( 2), the endemic equilibrium is obtained, thus proved.

Now, the basic reproduction number is obtained by employing the next-generation matrix method of Zhisheng and Van den Driessche [2012]. Thus;

$$R_{0}=\frac{\beta \;\Lambda \;\delta }{\mu \;k_{1}k_{2}}$$

### Local stability of equilibria

The stability of an equilibrium point is determined by considering all the roots of the characteristic equation of the Jacobian Matrix of model ( 1). If all the roots are negative then the equilibrium point is said to be locally asymptotically stable. The Jacobian Matrix J of the model is given as

$$J=\left[\begin{array}{lllll} -\beta I-\mu \; & 0\; & -\beta S\; & 0\; & 0\\ \beta I\; & -k_{1}\; & \beta S\; & 0\; & 0\\ 0\; & \delta \; & -k_{2}\; & 0\; & 0\\ 0\; & 0\; & \theta \; & -k_{3}\; & 0\\ 0\; & \tau_{1}\; & 0\; & \tau_{2}\; & -\mu \end{array}\right],$$

### Local stability of disease-free equilibrium point (ℰ _0_)

It can be shown that at $E_{}$, − *μ* and − *k*_3_ are roots of the characteristic equation of the Jacobian Matrix J. Clearly all these roots are negative. The roots that remain must therefore satisfy equation 3 at $E_{0}$. This is the characteristic polynomial of the Jacobian Matrix $J_{\in }$,

where

$$J_{\in }=\left[\begin{array}{ll} -k_{1} & \frac{\beta \;\Lambda }{\mu }\\ \delta & -k_{2} \end{array}\right]$$

The roots of equation 3 are the eigenvalues of $J_{\in }$.

$$\Phi_{2}x^{2}+\phi_{1}x+\phi_{0}=0$$ (3)

where

$$\left.\begin{array}{l} \Phi_{2}=1,\\ \Phi_{1}=k_{2}+k_{1}\\ \Phi_{0}=\left(1-R_{0}\right)k_{1}k_{2} \end{array}\right\}$$

Whenever $R_{0}<1$, all the coefficients Φ _*i*_, *i* = 0, 1, 2, are positive. This suggests lemma 2 by using Descartes rule of signs.

**Lemma 0.2.***The*$E_{0}$*is locally asymptotically stable whenever*$R_{0}<1$*and unstable whenever*$R_{0}>1$.

### Local stability of endemic equilibrium point (ℰ ^*^)

Again, the roots that remain must, after establishing that − *μ* and − *k*_3_ are roots of the characteristic equation of the Jacobian Matrix J, satisfy equation 4. This is the characteristic polynomial of the Jacobian Matrix $J_{∋}$ at $E^{*}$, where

$$J_{∋}=\left[\begin{array}{lll} -i\beta -\mu \; & 0\; & -\beta \;S\\ i\beta \; & -k_{1}\; & \beta \;S\\ 0\; & \delta \; & -k_{2} \end{array}\right]$$

The roots of equation 4 are the eigenvalues of $J_{∋}$.

$$\Pi_{3}x^{3}+\Pi_{2}x^{2}+\Pi_{1}x+\Pi_{0}=0$$ (4)

where

$$\left.\begin{array}{l} \Pi_{3}=1,\\ \Pi_{2}=k_{2}+k_{1}+\beta \;I+\mu \\ \Pi_{1}=\left(1-R_{0}\right)k_{2}k_{1}+k_{2}\beta \;I+k_{2}\mu +k_{1}\beta \;I+k_{1}\mu \\ \Pi_{0}=\left(1-R_{0}\right)k_{2}k_{1}\mu +k_{2}k_{1}\beta \;I \end{array}\right\}$$

Whenever $R_{0}\leq 1$, all the coefficients Π _*i*_, *i* = 0, 1, 2, 3 are positive. This also suggests lemma 3 by using Descartes rule of signs.

**Lemma 0.3.***The*$E^{*}$is locally asymptotically stable whenever$R_{0}\leq 1$*and unstable whenever*$R_{0}>1$.

### Global stability of disease-free equilibrium point (ℰ _0_)

The technique of Castillo-Chavez et al. [2002] is employed to study the global stability of $E_{0}$. If we let $Y=\left(S,R\right)$ and $X=\left(S_{1},Q,I,H\right)$, model ( 1) can now be re-written as

$$\left.\begin{array}{l} \frac{dY}{dt}=F(Y,X)\\ \frac{dX}{dt}=G(Y,X) \end{array}\right\}$$ (5)

where

$$F=\left[\begin{array}{l} \Lambda -\beta \;SI-\mu \;S\\ \tau_{1}E+\tau_{2}H-\mu R \end{array}\right]$$

and

$$G=\left[\begin{array}{lll} -k_{1}\; & \beta \;S\; & 0\\ \delta \; & -k_{2}\; & 0\\ 0\; & \theta \; & -k_{3} \end{array}\right].$$

According to Castillo-Chavez et al. [2002], the following conditions establish the global stability of $E_{0}$.

$$\begin{array}{l} H1:\text{For}\;{\left.\frac{dY}{dt}\right|}_{X=0},\;Y^{*}\;\text{ is globally asymptotically stable}\\ H2:G(Y,X)=LZ-\hat{G}(Y,X),\hat{G}(Y,X)\geq 0\;\text{for}\;(Y,X)\in D, \end{array}$$ (6)

where $L=D_{X}G(Y^{*},0)$ is the Jacobian of G(Y,X) with respect to *X* at $E_{0}$.

If the reduced system of ( 1) given by

$${\left.\frac{dY}{dt}\right|}_{X=0}=\left[\begin{array}{l} \Lambda -\mu \;S\\ -\mu \;R \end{array}\right]$$

is considered, then disease-free equilibrium point $Y^{*}=E_{0}$ is clearly a globally asymptotically stable point of the reduced system. Also, let $G(Y,X)=LX-\hat{G}(Y,X)$, where

$$L=D_{X}G(Y^{*},0)=\left[\begin{array}{lll} -k_{1}\; & \beta \;S\; & 0\\ \delta \; & -k_{2}\; & 0\\ 0\; & \theta \; & -k_{3} \end{array}\right]$$

and

$$\hat{G}(Y,X)=\lambda \;N\left[\begin{array}{l} \beta \;SI-k_{1}E\\ \delta \;E-k_{2}I\\ \theta \;I-k_{3}H \end{array}\right]$$

Substituting $E_{0}$, into $\hat{G}(Y,X)$ gives zero components and condition *H*_2_ is satisfied. Whenever $R_{0}\leq 1$ the $E_{0}$ is globally asymptotically stable hence establishing the following results.

**Lemma 0.4.***The disease-free equilibrium point*$E_{0}$*of the model is globally asymptotically stable whenever*$R_{0}\leq 1$.

### Global stability of endemic equilibrium point (ℰ ^*^)

In this section, we try to find out whether or not the endemic equilibrium is globally stable using Lyapunov functions technique.

**Theorem 0.2.***The endemic equilibrium of model (*1*) is globally asymptomatically stable whenever*$R_{0}>1$.

*Proof.* Consider the Lyapunov function

$$\begin{array}{ll} V= & \left(S-S^{*}-S^{*}\ln\frac{S^{*}}{S}\right)+\left(E-E^{*}-E^{*}\ln\frac{E^{*}}{E}\right)\\ & +\left(I-I^{*}-I^{*}\ln\frac{I^{*}}{I}\right)+\left(H-H^{*}-H^{*}\ln\frac{H^{*}}{H}\right)\\ & +\left(R-R^{*}-R^{*}\ln\frac{R^{*}}{R}\right). \end{array}$$

If we take the derivative of *V* we obtain

$$\begin{array}{ll} \frac{dV}{dt}= & \left(\frac{S-S^{*}}{S}\right)\frac{dS}{dt}+\left(\frac{E-E^{*}}{E}\right)\frac{dE}{dt}\\ & +\left(\frac{I-I^{*}}{I}\right)\frac{dI}{dt}+\left(\frac{H-H^{*}}{H}\right)\frac{dH}{dt}\\ & +\left(\frac{R-R^{*}}{R}\right)\frac{dR}{dt},\\ = & \left(\frac{S-S^{*}}{S}\right)\left[\Lambda +-\left(\beta \;I+\mu \right)\;S\right]\\ & +\left(\frac{E-E^{*}}{E}\right)\left[\beta \;S\;I-\left(\tau_{1}+\delta +\sigma +\mu \right)\;E\right]\\ & +\left(\frac{I-I^{*}}{I}\right)\left[\delta \;E-\left(\theta +\sigma +\mu \right)\;I\right]\\ & +\left(\frac{H-H^{*}}{H}\right)\left[\theta \;I-\left(\tau_{2}+\sigma +\mu \right)\;H\right]\\ & +\left(\frac{R-R^{*}}{R}\right)\left[\tau_{1}\;E+\tau_{2}\;H-\mu \;R\right]. \end{array}$$ (7)

After some algebraic manipulation $\frac{dV}{dt}=A-B$, where

$$\begin{array}{ll} A= & \frac{\left(S_{0}E-SE_{0}\right)I\beta }{E}+\frac{\delta \;E\left(I-I_{0}\right)}{I}+\frac{\theta \;I\left(H-H_{0}\right)}{H}+\frac{\tau_{1}E\left(R-R_{0}\right)}{R}+\frac{\tau_{2}H\left(R-R_{0}\right)}{R}, \end{array}$$

and

$$\begin{array}{ll} B= & \mu \;\left(S-S_{0}\right)+k_{1}\left(E-E_{0}\right)+k_{2}\left(I-I_{0}\right)+k_{3}\left(H-H_{0}\right)+\mu \;\left(R-R_{0}\right). \end{array}$$

Therefore whenever $A<B\Rightarrow \;\frac{dV}{dt}<0,$ and also $A-B=0\Rightarrow \;\frac{dV}{dt}=0,$ this is possible if *S* = *S* ^*^, *E* = *E* ^*^, *I* = *I* ^*^, *H* = *H* ^*^, *R* = *R* ^*^. Therefore, the largest compact invariant set for model ( 1) is $\left\{E^{*}\right\}$. Hence the global asymptotic stability of $E^{*}$ is established in the positive region $R_{\geq 0}^{5}$ if **A** < **B** for $R_{0}>1$ as in Lyapunov-LaSalle’s stability theorem [ LaSalle, 1968],

### Sensitivity analysis of the reproduction number (ℛ _0_)

Parameters play a very important role in the dynamical behaviour of models; therefore the study of the impact of changes in the values of these parameters cannot be underestimated [ Seidu et al., 2020; Bornaa et al., 2021; Chitnis et al., 2008]. The influential parameters of the model are considered by beginning to value the changes that occur in their values. Sensitivity analysis is therefore employed to help identify such influential parameters of the model. The normalized forward sensitivity technique is employed to study the sensitivity of $R_{0}$ to model parameters.

**Definition:** Let model output $R_{0}$ be differentiably dependent on model parameter *x.* The normalized forward sensitivity index of $R_{0}$ with respect to *x* is defined by

$${ϒ}_{x_{i}}^{R_{0}}={\left.\frac{\partial R_{0}}{\partial x_{i}}\times \frac{x_{i}}{R_{0}}\right|}_{\left(x_{1},\;x_{2},\;\ldots ,x_{n}\right)}$$ (8)

The model parameter values in Table 1 are carefully selected as baseline values for the purposes of the sensitivity test and numerical simulation only. The local sensitivity indexes are therefore recorded in Table 2 taking $R_{0}$ as a model output.

**Table 1.** Descriptions and baseline values of model parameters.

Par.	Description	Value
*μ*	Natural death rate	0.01
*Λ*	Rate of recruitment	20
*τ*_1_	Rate of early treatment	0.5
*β*	Transmission probability	0.002
*σ*	COVID-19 induced death rate	0.02
*θ*	Rate of hospitalization of infected persons	0.3
*τ*_2_	Recovery rate of treated infected individuals	0.2
*δ*	Rate at which latently infected indivduals join the infected class	0.0012

**Table 2.** Sensitivity Indexes.

Parameters	*β*	Λ	*δ*	*μ*
Sensitivity Index ( ${ϒ}_{x_{i}}^{R_{0}}$)	1.0000	1.0000	0.9977	-1.0491
Parameters	*τ*_1_	*θ*	*σ*	
Sensitivity Index ( ${ϒ}_{x_{i}}^{R_{0}}$)	-0.9413	-0.9091	-0.0983	

## Numerical simulation

Considering the baseline parameter values in Table 1, numerical experiments are performed, with initial values of *S* = 0.411 *e* + 5, *E* = 0.493 *e* + 2, *I* = 0.243 *e* + 2, *H* = 0.318 *e* + 2, *R* = 0.97 *e* + 3, from literature to verify the analytical results established. The simulation is carried out using matlab version 1.0 for MathWorks R2017a release.

Simulation to illustrate the local stability of the disease free equilibrium of model ( 1) is run for $R_{0}<1$.

Figure 2 depicts the bar graph of the sensitivity of *R*_0_ to marginal changes in each of the parameters that constitute *R*_0_.

**Figure 1.**
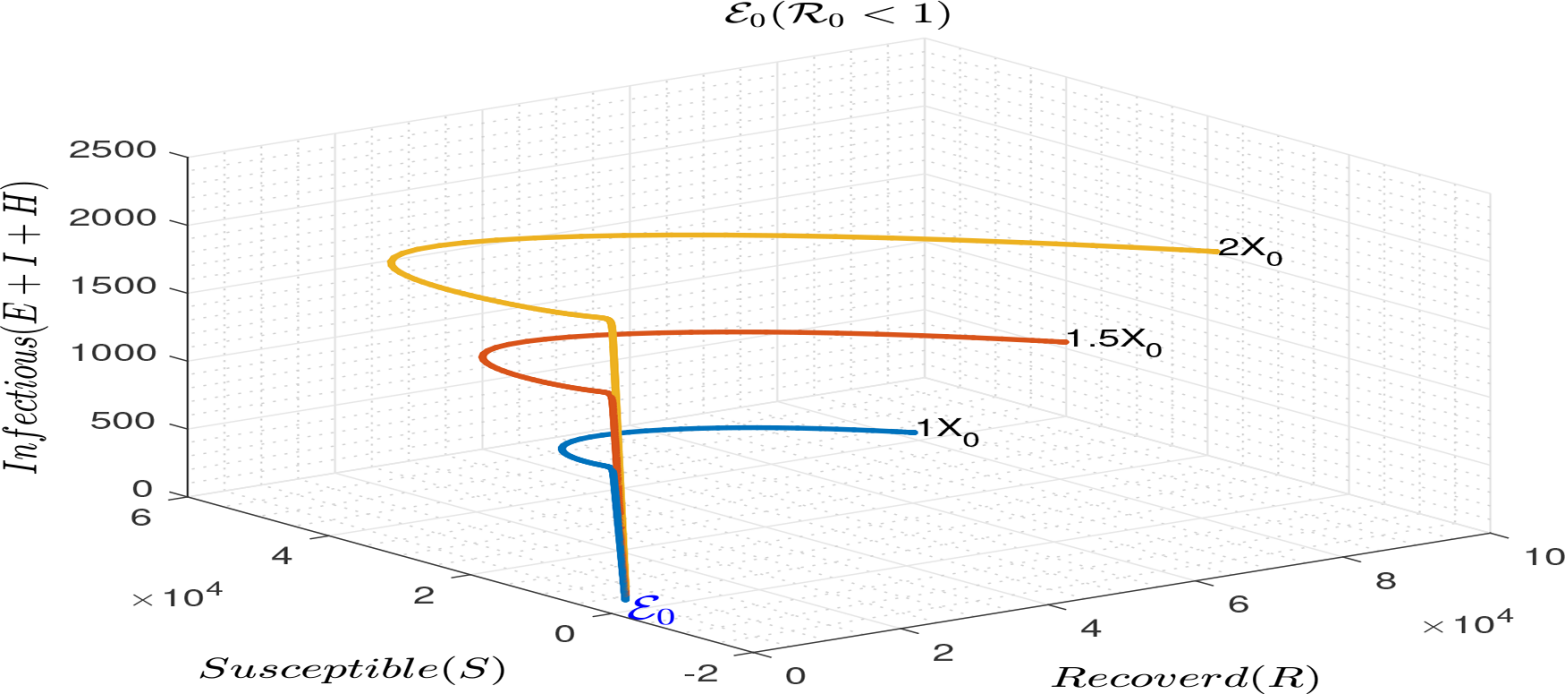
Simulation results illustrating the local stability of ℰ _0_ for ℛ _0_ < 1.

**Figure 2.**
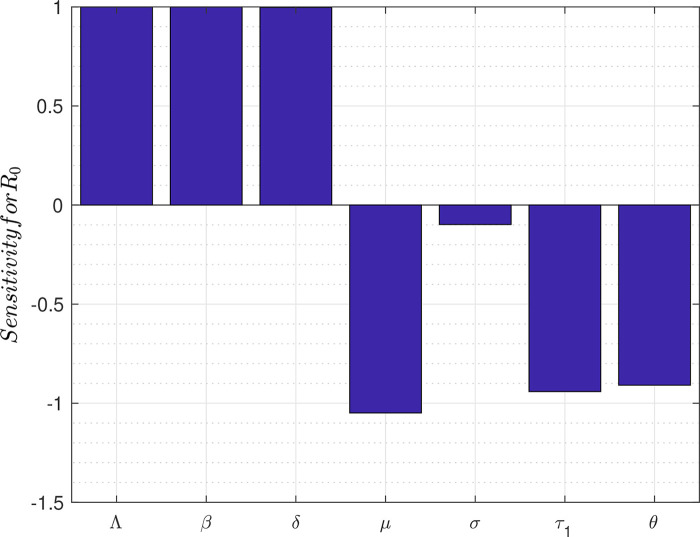
Plot for sensitivity of parameters of *R*_0_.

To illustrate the impact of early treatment on the dynamics of coronavirus infection, we now present the early treatment parameter *τ*_1_, which is varied (from initial value of 0.5 at 0.5 intervals) and observe its impact on the spread of coronavirus infection, with all other parameters kept constant (see Figure 4 *a*, *b*, *c*, *d* and *e*).

**Figure 3.**
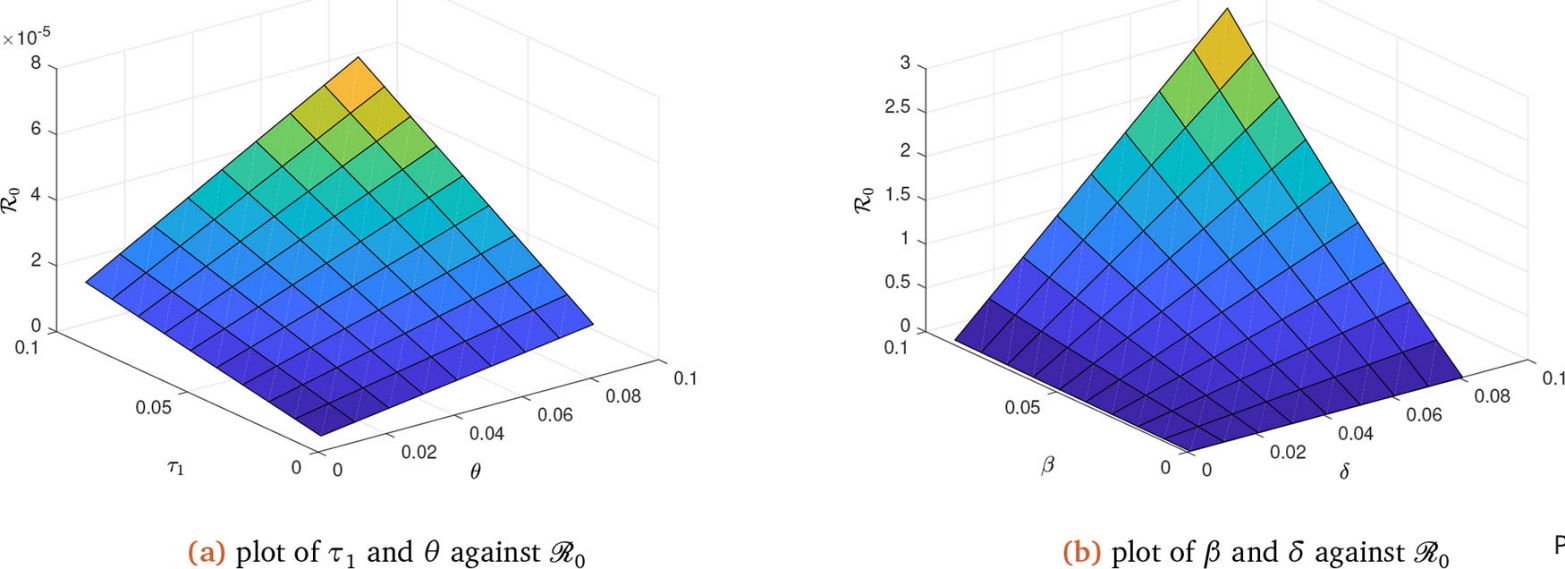
Solution curves depicting the impact of *τ*_1_, *θ*, *β* and *δ* on ℛ _0_.

**Figure 4.**
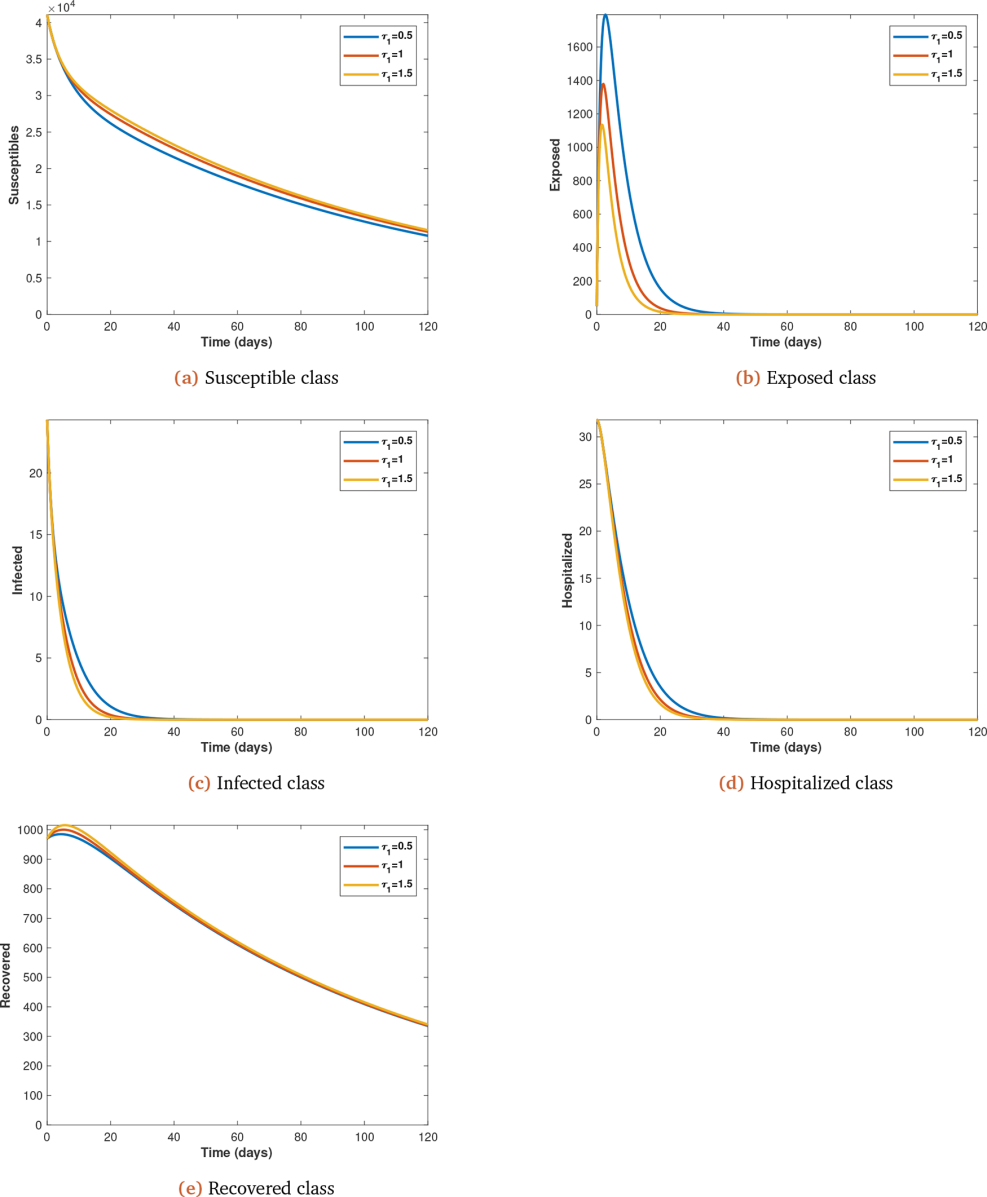
Solution curves depicting the impact of *τ*_1_ on each of the classes.

**Figure 5.**
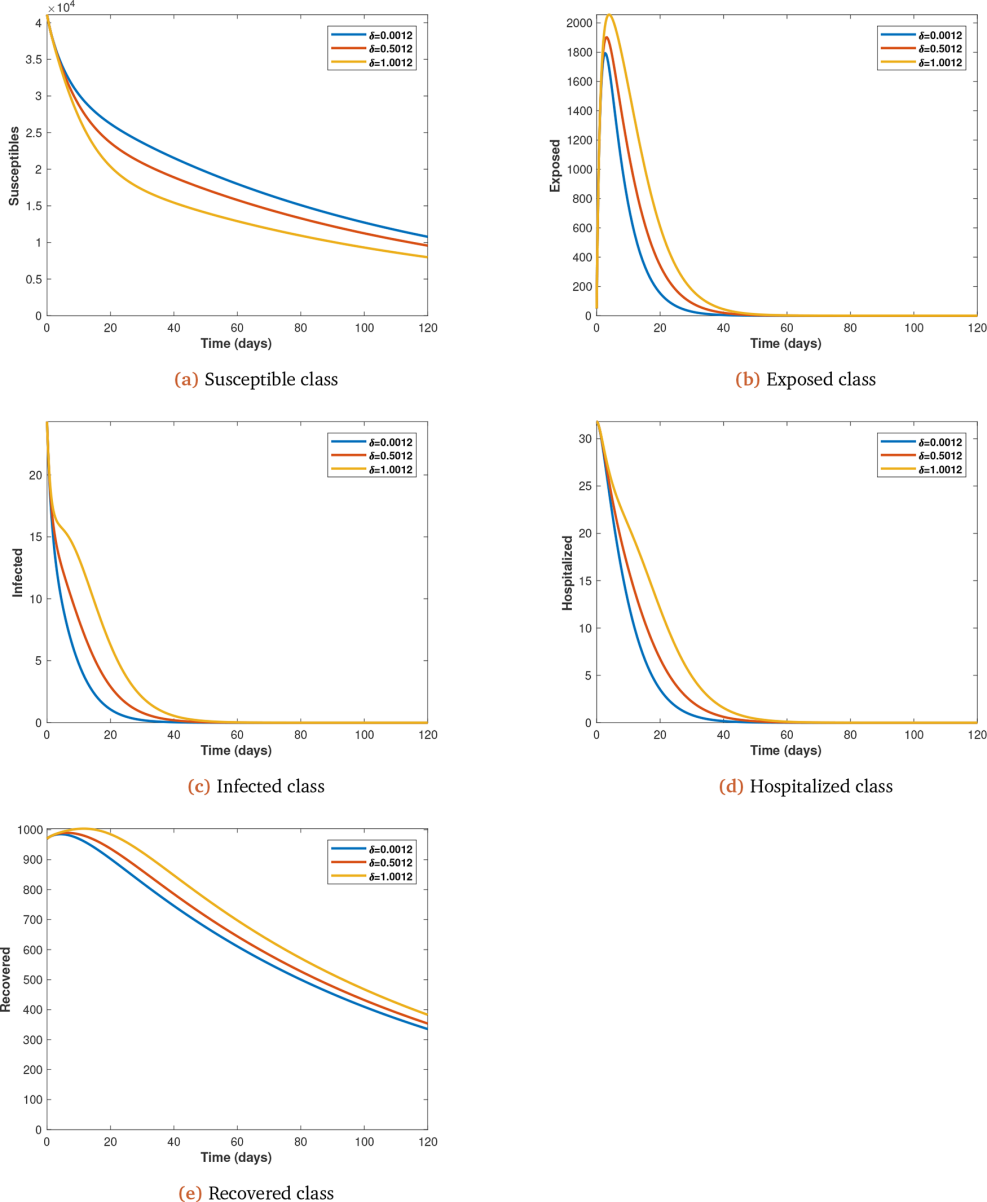
Solution curves depicting the impact of *δ* on each of the classes varied from initial value of 0.0012 at 0.5 intervals.

**Figure 6.**
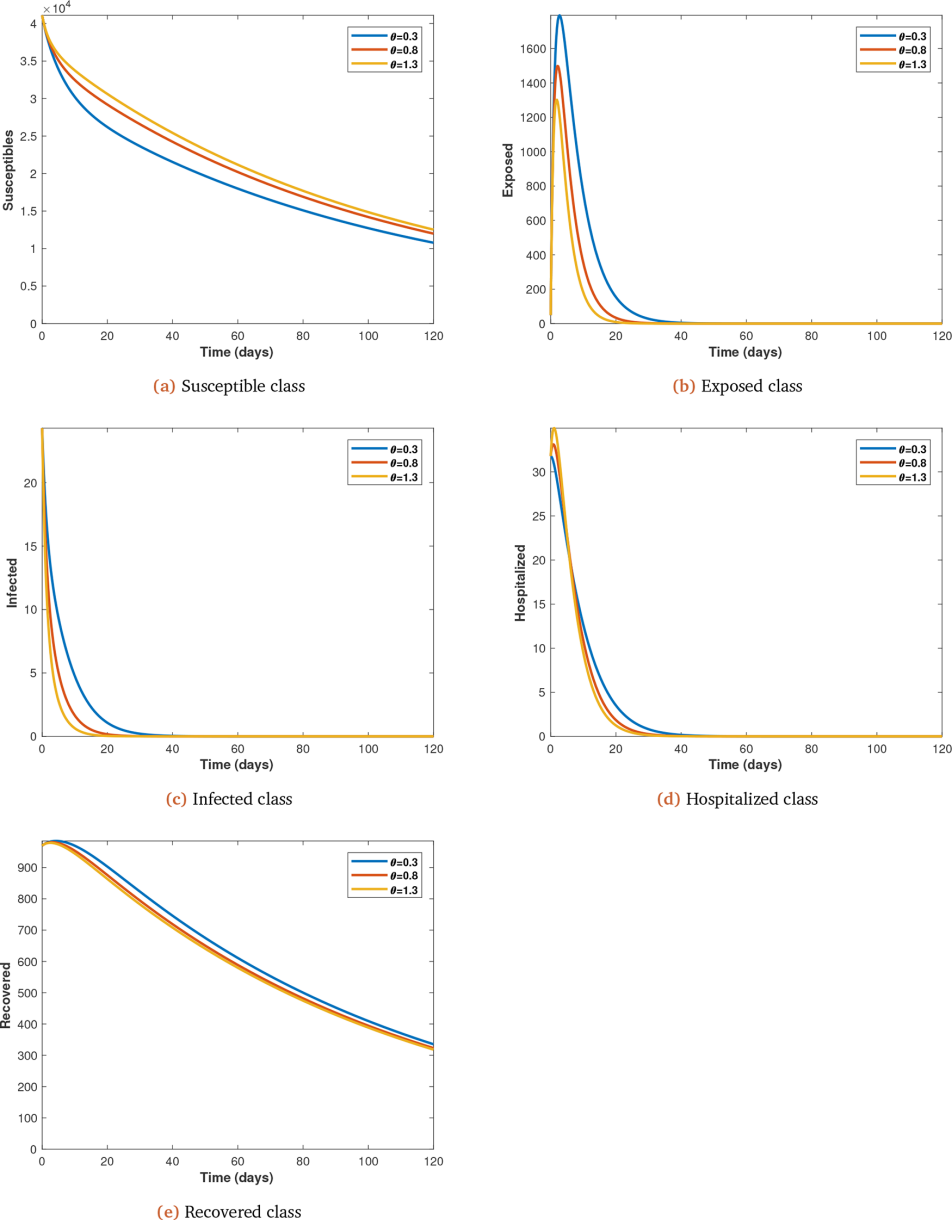
Solution curves depicting the impact of *θ* on each of the classes varied from initial value of 0.3 at 0.5 intervals.

**Figure 7.**
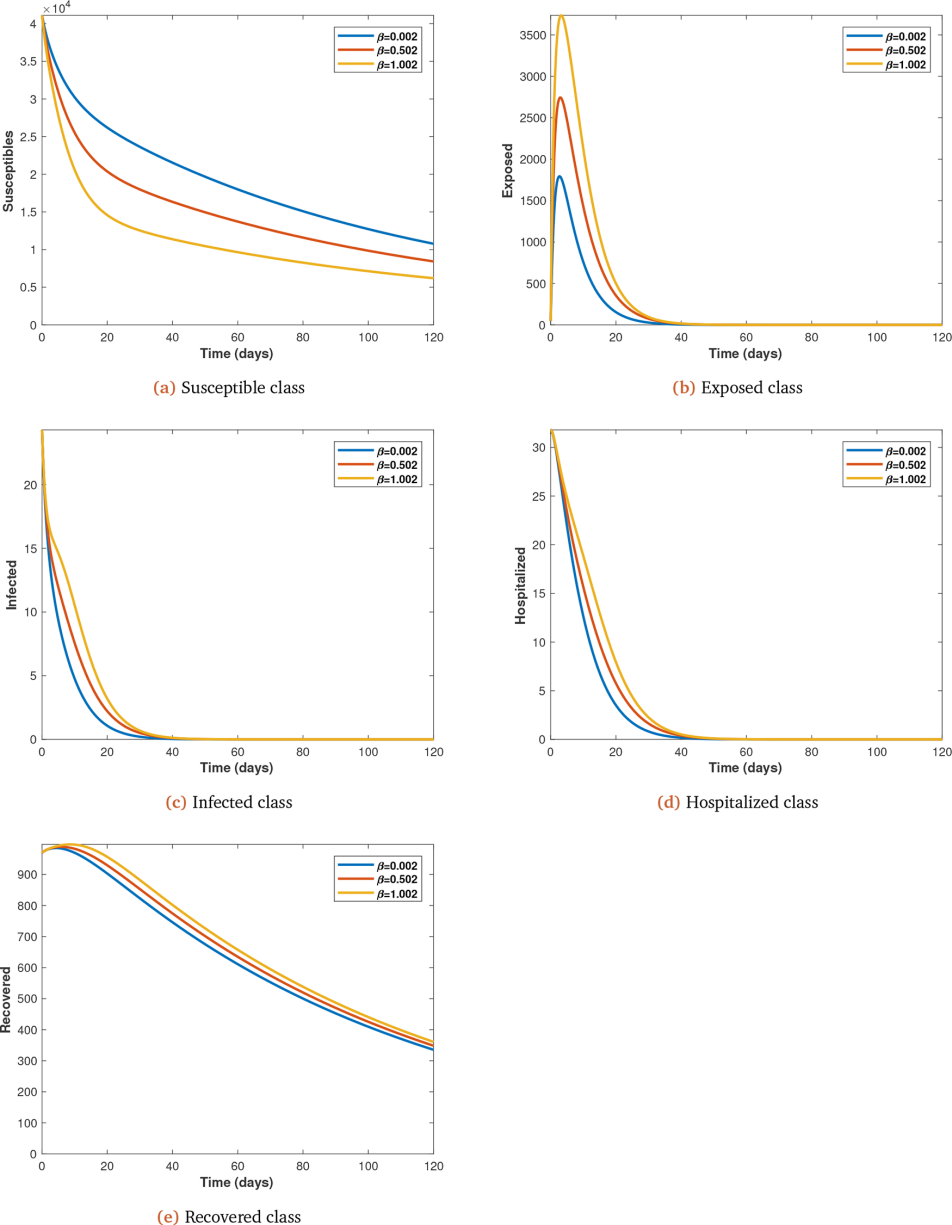
Solution curves depicting the impact of *β* on each of the classes varied from initial value of 0.002 at 0.5 intervals.

Each parameter under consideration is varied (from initial value at 0.5 intervals) to observe its impact on the spread of Covid-19 infections, with all other parameters kept constant (see Figure 5 *a*, *b*, *c*, *d* and *e* for *δ*; Figure 6 *a*, *b*, *c*, *d* and *e* for *θ*; Figure 7 *a*, *b*, *c*, *d* and *e* for *β*).

## Discussions, conclusions and recommendations

A deterministic model is proposed to describe the transmission dynamics of coronavirus infection with early interventions. The study describes the basic qualitative properties of the model and carried out numerical simulations to confirm the qualitative results. The model is shown to have unique disease-free and endemic equilibria (see equation 2, theorem 1 and the proof). The disease-free equilibrium is locally asymptotically stable when $R_{0}<1$ (see Figure 1). The endemic equilibrium is also locally asymptotically stable when $R_{0}\leq 1$. The global stability of the disease-free and endemic equilibria are respectively established under the conditions; $R_{0}\leq 1$ and $R_{0}>1$.

Analysis of the responsiveness of the model parameters to marginal changes is also carried out to identify the most important factors to consider in the fight against the spread of the disease. The results show, in order of importance, that a marginal change in any of the following parameters will affect directly or inversely the reproduction number thereby affecting the transmission dynamics of the disease. These parameters are: the death rate *μ*, transmission probability *β*, recruitment rate *Λ*, rate at which exposed individuals join the infected class *δ*, early treatment rate *τ*_1_, rate of hospitalization of infected individuals *θ* and coronavirus induced death rate *σ* (see Table 2).

We observe from Table 2 that *Λ*, *β* and *δ* are directly proportional to $R_{0}$ in order of importance. In other words, an increase (decrease) in any of these parameters will lead to an increase (decrease) in $R_{0}$ as confirmed by Figure 3 *b* on *β* and *δ.* The parameters that are however inversely proportional to $R_{0}$ are *μ*, *θ*, *τ*_1_ and *σ.* An increase (decrease) in any of these parameters will lead to a decrease (increase) in $R_{0}$ as confirmed by Figure 3 *a* on *τ*_1_ and *θ.* This is also reflected in Figure 4 ( *a*, *b*, *c*, *d* and *e*). Increasing early treatment reduces the infected population (see Figure 4 *b*, *c* and *d*) and increases the non-infected population (see Figure 4 *a* and *e*). This is also supported by Kim et al. [2020] and Strain et al. [2005] when they carried out independent studies into early treatment of infectious diseases.

Many health care facilities are overstretched in terms of space, equipment and personnel because of the pandemic. This challenge can be handled by either providing more space, equipment and personnel, or by increasing the recovery rate of treated infected individuals. This study has confirmed that an increase in recovery rate reduces the hopitalized individuals and thereby reduces the pressure on health care facilities to take in more coronavirus and other patients (see Figure 6d).

It is assumed that the recovered gained permanent immunity because they develop antibodies that react and fight against initial infections. We also observe increases in the rate at which the exposed joins the infected (symptomatic) class ( *δ*). When this happens the symptomatic are moved into hospitals for treatment and subsequently recovery. The effect is that the infected and recovered populations are increased but the susceptible population is reduced because of the immunity gained by the recovered (see Figure 5 *a*, *b*, *c*, *d* and *e*).

It is also shown that as transmission probability increases the infected increase and the susceptible decrease (see Figure 7 *a*, *b*, *c* and *d*).

It is recommended that:

• early treatment of coronavirus should be considered. This can be effectively carried out when proper surveillance is enforced to identify asymptomatic individuals who are mostly at their early stage of infection. People should also be encouraged to use immune boosters to reduce the rate at which the exposed joins the infected while early treatment is enforced.
• efforts should be made to reduce the rate of infection by insisting on people observing all the coronavirus protocols announced by World Health Organisation and other health institutions.

## Data availability

All data underlying the results are available as part of the article and no additional source data are required.

## Author contributions

• Christopher Saaha Bornaa: Conceptualization and algebriac analysis
• Baba Seidu: Numerical analysis
• Yakubu Ibrahim Seini: Proof reading, supervision and advisory role
