# Supplementary material for: Modeling the impact of early interventions on the transmission dynamics of coronavirus infection
Source: F1000Res. 2021 Aug 17;10:518. Originally published 2021 Jun 30. [Version 2] doi: 10.12688/f1000research.54268.2 (PMC8411273; doi:10.12688/f1000research.54268.2)
Supplement: Peer review history

# Peer review: Reviewer response for version 2

**DOI:** 10.5256/f1000research.76442.r92124

**Published:** 2021-09-01

**Authors:** Mehmet Yavuz

**Affiliations:** 1 Department of Mathematics and Computer Sciences, Faculty of Science, Necmettin Erbakan University, Konya, Turkey; 2 Department of Mathematics, College of Engineering, Mathematics and Physical Sciences, University of Exeter, Cornwall, TR10, UK

The revised version of the paper has been properly prepared. It can be indexed as it is.

Is the rationale for developing the new method (or application) clearly explained?

Partly

Is the description of the method technically sound?

Yes

Are the conclusions about the method and its performance adequately supported by the findings presented in the article?

Partly

If any results are presented, are all the source data underlying the results available to ensure full reproducibility?

Partly

Are sufficient details provided to allow replication of the method development and its use by others?

Yes

Reviewer Expertise:

Mathematical modelling in epidemiology, fractional calculus, applied mathematics, optimal control, bifurcation and chaos.

I confirm that I have read this submission and believe that I have an appropriate level of expertise to confirm that it is of an acceptable scientific standard.

# Peer review: Reviewer response for version 1

**DOI:** 10.5256/f1000research.57739.r89506

**Published:** 2021-08-25

**Authors:** Ebenezer Bonyah

**Affiliations:** 1 Department of Mathematics Education, Akenten Appiah-Menka University of Skills Training and Entrepreneurial Development, Kumasi, Ghana

- The authors appreciate that there are many mathematical models available on COVID-19.
- The authors must provide the knowledge gap that this work is filling. 
- The authors must present anything new on the model formulated.
- The authors must also provide a reason for using this numerical scheme for this study.
- For the conclusions, I suggest for this to be in the past tense.

Is the rationale for developing the new method (or application) clearly explained?

Yes

Is the description of the method technically sound?

Yes

Are the conclusions about the method and its performance adequately supported by the findings presented in the article?

Yes

If any results are presented, are all the source data underlying the results available to ensure full reproducibility?

Yes

Are sufficient details provided to allow replication of the method development and its use by others?

Partly

Reviewer Expertise:

Mathematical Biology, Mathematical Modelling and Mathematics Education

I confirm that I have read this submission and believe that I have an appropriate level of expertise to confirm that it is of an acceptable scientific standard.

# Peer review: Reviewer response for version 1

**DOI:** 10.5256/f1000research.57739.r88778

**Published:** 2021-07-26

**Authors:** Mehmet Yavuz

**Affiliations:** 1 Department of Mathematics and Computer Sciences, Faculty of Science, Necmettin Erbakan University, Konya, Turkey; 2 Department of Mathematics, College of Engineering, Mathematics and Physical Sciences, University of Exeter, Cornwall, TR10, UK

1. Authors should put proper punctuation at the end of all equations.
2.  In numerical simulations, the authors need to explain where they have taken the parameter values from. Are they from the literature or estimated ones?
3.  What makes the proposed methods and COVID-19 model suitable for this unique task? What new development to the proposed model have the authors added (compared to the existing approaches)? These points should be clarified.
4. What is the novelty of your work? There are some similar papers that have been investigated in epidemiology, especially COVID-19, although there are some minor differences in the structure of models. Please state it clearly.
5. Have you employed any assumptions in your COVID-19 model and problem formulation? Please explain briefly.
6. More physical interpretations should be given. Please provide corresponding explanations of the figures in terms of their physical meanings and pointing out the novelty of the paper. How do figures support your scheme?
7. The authors should improve the introduction by including the recent development within the frame of the mentioned COVID-19 models. Also, their mathematical investigations and numerical simulations with the help of recently published papers should be considered by comparing their current model. Sensitivity analysis of the Reproduction number can be discussed deeply. In this connection, I suggest some interesting results:

- Modeling and analysis of COVID-19 epidemics with treatment in fractional derivatives using real data from Pakistan ^1^.
- A New Mathematical Modeling of the COVID-19 Pandemic Including the Vaccination Campaign. Open Journal of Modelling and Simulation ^2^.
- New Approaches to the Fractional Dynamics of Schistosomiasis Disease Model. Physica A: Statistical Mechanics and its Applications ^3^.

Is the rationale for developing the new method (or application) clearly explained?

Partly

Is the description of the method technically sound?

Yes

Are the conclusions about the method and its performance adequately supported by the findings presented in the article?

Partly

If any results are presented, are all the source data underlying the results available to ensure full reproducibility?

Partly

Are sufficient details provided to allow replication of the method development and its use by others?

Yes

Reviewer Expertise:

Mathematical modelling in epidemiology, fractional calculus, applied mathematics, optimal control, bifurcation and chaos.

I confirm that I have read this submission and believe that I have an appropriate level of expertise to confirm that it is of an acceptable scientific standard, however I have significant reservations, as outlined above.

## References

[1] : Modeling and analysis of COVID-19 epidemics with treatment in fractional derivatives using real data from Pakistan.*Eur Phys J Plus*.2020;135(10) : 10.1140/epjp/s13360-020-00819-579510.1140/epjp/s13360-020-00819-533145145PMC7594999

[2] : A New Mathematical Modeling of the COVID-19 Pandemic Including the Vaccination Campaign. *Open Journal of Modelling and Simulation*.2021;09(03) : 10.4236/ojmsi.2021.93020299-32110.4236/ojmsi.2021.93020

[3] : New approaches to the fractional dynamics of schistosomiasis disease model. *Physica A: Statistical Mechanics and its Applications*.2019;525: 10.1016/j.physa.2019.03.069373-39310.1016/j.physa.2019.03.069
